# Early Supported Discharge for Neurorehabilitation Following Acquired Brain Injury

**DOI:** 10.3389/fneur.2020.596526

**Published:** 2020-11-30

**Authors:** Regan King, Trevor Seeger, Meng Wang, Rodney Li Pi Shan, Christine McGovern, Jason Knox, Lisa Patel, Tak Fung, Tolulope Sajobi, Chantel Debert

**Affiliations:** ^1^Calgary Brain Injury Program, Alberta Health Services, Calgary, AB, Canada; ^2^Department of Clinical Neurosciences, Cumming School of Medicine, University of Calgary, Calgary, AB, Canada; ^3^Department of Community Health Sciences, University of Calgary, Calgary, AB, Canada; ^4^Department of Nursing, University of Calgary, Calgary, AB, Canada

**Keywords:** early supported discharge (ESD), neurorehabiliation, acquired brain injured (ABI), caregiver burden, functional outcome, in-home rehabilitation

## Abstract

**Introduction:** Early Supported Discharge (ESD) is a clinical flow management service offering interdisciplinary rehabilitation, wherein patients are provided supported in-home rehabilitation treatment; in comparison to conventional hospital-based rehabilitation model of service delivery. There has been little research into the functional outcomes for other types of acquired brain injury (ABI).

**Methods:** In this prospective cohort study, ABI patients presenting at a level I trauma center in Calgary, Canada were placed in either an ESD program or conventional inpatient rehabilitation (IPR) program based on their medical history and presentation. A small number of patients completed both programs (ESD+IPR group). ESD therapies were designed to emulate IPR. Participants completed professionally-rated Mayo-Portland Adaptability Index-4 (MPAI), Quality of Life after Brain Injury (QOLIBRI), Generalized Anxiety Questionnaire-7 (GAD7), Montreal Cognitive Assessment (MoCA), and Patient Health Questionnaire-9 (PHQ9) surveys at 1, 3, and 6 months following initial assessment pre-rehabilitation. Caregivers completed the Zarit Burden Interview (ZBI) at the same time points. The Supervision Rating Scale (SRS) and Disability Rating Scale (DRS) were completed at admission to rehabilitation and all follow-ups. Generalized estimate equations models were used to describe the three groups over time, including age as a covariate.

**Results:** Significant effects of time were reported in the MPAI participant sub-score in the ESD and IPR groups ($\chi_{\left(2\right)}^{2}$ = 42.429, *p* < 0.000; $\chi_{\left(2\right)}^{2}$ = 9.773, *p* = 0.008), showing significantly higher scores between 1 and 3 month timepoints for both groups. ZBI scores were significantly lower in the ESD group at 1 month compared to 3 and 6 months ($\chi_{\left(2\right)}^{2}$ = 31.252, *p* < 0.001). The proportion of patients with medical complications during rehabilitation was 25.3% in ESD compared to 74.7% patients in IPR.

**Conclusions:** Improvements in functional outcomes were evident in patients participating in ESD and IPR, with more medical complications reported in the IPR group. Caregiver burden lessened over time in the ESD group but not in the IPR group. Both ESD and ESD+IPR groups can be considered viable alternatives to traditional inpatient rehabilitation. A randomized control trial would be required to properly compare rehabilitation streams. Further investigation into affective and lifestyle elements of ABI recovery would also improve our understanding of targeted neurorehabilitation in this population.

## Introduction

Recovery from acquired brain injury (ABI) can be as varied as the injuries themselves. Symptoms vary widely and may involve physical symptoms such as motor or speech deficits, but may also involve affective and personality changes such as aggression or anxiety (1–3). Most commonly, traditional neurorehabilitation includes transition from acute care to rehabilitation as an inpatient at a tertiary care center; involving a multidisciplinary health care team to address the personalized rehabilitation needs. Inpatient rehabilitation (IPR) is focused on providing daily intensive rehabilitation to improve function by providing physiotherapy, occupational therapy, recreational therapy, nursing, speech language pathology, and education; as well as social work a psychology services. Patients are required to stay in hospital for the duration of the required rehabilitation and upon discharge at our center there is often a lengthy wait to participate in outpatient rehabilitation.

In contrast, early supported discharge (ESD) involves implementing a neurorehabilitation program for medically stable patients to complete in their home, to replace traditional in-hospital neurorehabilitation. Patients approved for ESD are supported by neurorehabilitation teams that provide individualized therapies in a familiar environment. Individuals can work toward remediating daily living skills while re-adapting to their home environment. It is suggested that the personalized physical and social atmosphere of the home facilitates relearning of daily living skills more effectively than that of the hospital (4). Additionally, ESD programs are thought to be more cost-effective for national health care systems as they reduce burden on inpatient programs while promoting the patient's own return to independence (3, 5).

Various ESD rehabilitation programs have been explored in individuals with stroke (4, 6, 7), and successfully been implemented as a treatment option in Calgary, Canada (5). Findings from ESD programs with individuals with stroke suggest that at-home therapies can improve independence, and skills for daily living activities (8–10). Greater patient satisfaction has also been reported following participation of patients with stroke in ESD programs (4, 6). A longitudinal, randomized control trial found that patients with stroke receiving in-home rehabilitation compared to those receiving inpatient rehabilitation were more independent in extended activities of daily living and motor capacity (10). While literature from individuals with stroke provides an applicable model for acquired brain injury, very few studies have evaluated ESD rehabilitation in non-stroke ABI populations. A pilot study conducted by Doig et al. (11) evaluated the effectiveness of inpatient vs. at-home rehabilitation in patients with TBI and did not find any significant differences. Another study by the same group found that participants recovering from TBI who underwent treatment in both inpatient and at-home settings preferred the at-home treatment (12). There is a distinct lack of literature evaluating the effectiveness of at-home therapies in the non-stroke ABI population. Further exploration is needed to determine if ESD is a viable, long term therapeutic option for recovery from ABI.

With this knowledge, we aimed to describe and evaluate the efficacy of an ESD program for non-stroke ABI patients. Further, we aimed to characterize longitudinal recovery of these patients, in both IPR and ESD programs. We hypothesized that an ESD program for medically-stable patients with ABI could provide intensive comprehensive rehabilitation in the community to improve functional, psychological, and caregiver outcomes similar to inpatient rehabilitation.

## Materials and Methods

### Enrolment

For this prospective cohort study, patients with non-stroke ABI were selected from the acute care medical units at Foothills Medical Center, a level I trauma center in Calgary, Alberta, Canada from April 2016 to 2017. Written informed consent was obtained from all study participants prior to any data collection. If the participant was unable to give consent, surrogate consent was sought. This study was approved by the University of Calgary Conjoint Health Research Ethics Board and the REB# 16-0573.

#### Eligibility

Following injury, patients were treated in acute care until they were medically stable to begin intensive neurorehabilitation. The appropriate rehabilitation stream was determined for each patient by the attending brain injury Physical Medicine and Rehabilitation (PMR) physician and team, based on the following criteria: (1) were diagnosed with an ABI, excluding stroke, (2) were medically stable, (3) were able to participate in two different modalities of rehabilitation for a total of 60 min daily (4) gave written informed consent.

Considerations for individuals who were deemed appropriate candidates for the ESD program included the following: (1) safe for discharge and did not require the rehabilitation staff to be there for safety, (2) safe for independent transfer or with 1 person assist, (3) had necessary equipment in place in the home (e.g., walker, bath seat), (4) rehabilitation goals would be best served in the patient's own home environment to ensure contextually based therapy was delivered, (5) patient demonstrated cognitive competence and ability to carry-over new information (e.g., patient is not in post-traumatic amnesia), (9) tolerant of daily rehabilitation (up to 15 h/week), (10) and the patient consented to participate in ESD prior to discharge from hospital. In the above inclusion criteria, safe for discharge meant that the patient was sufficiently independent to be home alone [Supervision Rating Scale (SRS) score 1 or 2], or if supervision was required (SRS score 3–10) (13), family or caregiver was able to meet long-term supervisory needs without risking caregiver burnout. Patients were excluded if they received private rehabilitation outside of Alberta Health Services-funded rehabilitation or lived outside the Calgary city limits. They were also excluded if they exhibited behaviors or psychosocial concerns that limited their ability to participate in rehabilitation.

Individuals who participated in the IPR group typically had more severe rehabilitation needs, failing to satisfy the above inclusion criteria. Additionally, patients in IPR may have had other medical concerns impacting their stability for discharge.

#### Early Supported Discharge

Patients participating in ESD were seen 3–5 days per week receiving ~2–3 h per day of therapy, in their homes. The inter-professional team consisted of physiotherapy, neuropsychology, occupational therapy, recreational therapy, social work, psychology, and speech language pathology. Health care professionals involved in ESD care are paid using the same pay-scale as those who work for inpatient programs. A case manager and physical medicine and rehabilitation physician were assigned to monitor the patient's progress and ensure their continued safety. Rehabilitation programs were specified to the patient's individual needs.

#### Inpatient Rehabilitation

Patients participating in IPR were seen 3–5 days a week receiving 2–3 h of therapy daily within the hospital setting. Members of the rehabilitation team included physiotherapy, occupational therapy, recreational therapy, social work, psychology, speech language pathology, nursing, a physical medicine and rehabilitation physician, and a family physician. IPR programs were designed to target patients' individual needs.

### Study Procedures

Patients were assigned to IPR and ESD groups at the discrepancy of the attending physician to best address each patient's specific rehabilitation needs. A group of patients who participated in both ESD and IPR programs were also included in this study. Patients were assessed prior to admission to rehabilitation, then at 1, 3, and 6 months after consenting to the study. At initial assessment, patient characteristics, past medical history, cognitive performance (MoCA) (14), SRS (13), and Disability Rating Scale (DRS) (15) were completed. During follow-up sessions at 1, 3, and 6 months the addition of Quality of Life after Brain Injury (QOLIBRI) (16) for the participants, the Professionally-rated Mayo Portland Adaptability Index-4 (MPAI) (17) completed by staff and the Zarit Burden Interview (ZBI) (18) for the caregivers and family were included. Pre-existing co-morbidities and medical complications during rehabilitation were collected from the patients' medical records.

### Instruments

#### Mayo-Portland Adaptability Inventory–4

The professionally-rated MPAI and its sub-scales were collectively chosen as the primary outcome measure. The MPAI is a reliable measure, with satisfactory internal consistency and inter-rater reliability across rating sources (professional, significant other relation, person with injury, etc.) (19). The MPAI sub-scores comprise participation, ability, and adjustment. The ability sub-score includes 13 items relating to physical and cognitive capabilities, and the adjustment sub-score includes 12 items relating to emotional, psychological, and behavioral aspects of the patient's life. The participation subscale describes the return to normal activities (driving, employment, socializing, house maintenance, etc.) using eight questions. The participation subscale particularly, has been used in in-home and community-based rehabilitation of traumatic brain injury (20). The questions in the MPAI are scored on a 5-point scale from 0 to 4, where: 0 indicates that there are no problems or limitations in that aspect of the patient's life and 4 indicates that a severe problem is present (19).

#### Montreal Cognitive Assessment

The MoCA is a brief measure used to screen for cognitive impairment in a clinical population (14). The MoCA is scored out of 30, with a score above 26/30 considered normal. Scores below 26 indicate a degree of cognitive impairment.

#### Generalized Anxiety Disorder Questionnaire–7

The GAD7 measures severity of anxiety as a rapid screening tool for clinically significant anxiety (21). This 7-item measure has good reliability and procedural validity. Scores are reported as a frequency of symptoms in the past 14 days, with scores <5 indicating mild anxiety, scores between 5 and 10 indicating moderate anxiety, and scores >15 indicating severe feelings of anxiety.

#### Patient Health Questionnaire–9

The PHQ9 objectifies depression severity in patients. This 9-question tool incorporates the DSM IV criteria for depression into a simple, self-report tool (22). As with the GAD 7, scores are reported as a frequency of symptoms in the past 14 days. Scores below 4 indicate mild or absent depression, scores between 4 and 14 indicate moderate depression, while scores above 15 indicate severely depressed mood in need of further medical attention.

#### Quality of Life in Persons With Brain Injury Inventory

The QOLIBRI is a 38-item questionnaire (16) measuring health-related quality of life specific to TBI, and is recommended over generic measurements of health-related quality of life. Participants are asked to complete 28 questions asking how satisfied they are with cognitive, emotional, ADL, and social factors on a scale from “Not at all Satisfied” (0) to “Very Satisfied” (5). The second portion of the questionnaire uses a reversed version of the same scale, asking participants how bothered they are by emotional and physical problems (“Bothered” replacing the word “Satisfied”). Test-retest reliability is above 0.73 for all scales (16).

#### Supervision Rating Scale

The Supervision rating scale (SRS) (13) is a 13-point ordinal scale used to describe the level of supervision a person requires. A low score indicates relative independence while a high score indicates increased need for supervision and safety precautions.

#### Disability Rating Scale

The disability rating scale (DRS) (15) rates the level of cognitive impairment in different activities of daily life (ADLs), including eye opening, communication, grooming, toileting, feeding, bathing, up to their ability to be employed. It specifies that these questions pertain to the participants' cognitive capabilities, not their physical ability to perform the ADLs.

#### Zarit Burden Interview

The Zarit Burden Interview (ZBI) (18) is a caregiver self-report measure that addresses personal strain and role strain. It is not influenced by age, gender, locale, language, living situation, marital status, or employment status and therefore is suggested to be a useful measurement of caregiver burden in many different disease processes.

### Data Processing and Statistics

Data were entered into a secure online Research Electronic Data Capture (Redcap; Nashville, TN. Version 7.6.9) platform (23) hosted at the University of Calgary.

Due to correlated and unbalanced nature of the data, statistical comparisons were performed using generalized estimating equations (GEE) to detect significant time effect controlling for age. Significant comparisons were evaluated *post-hoc* using Bonferroni corrections. Categorical variables were compared independently, evaluating change within groups, longitudinally. All continuous descriptive variables were tested with age as a covariate. Chi-squared tests were used to compare demographic and injury characteristics between groups. Statistical analyses were conducted using SPSS V23.0 (SPSS Inc., Chicago, Illinois). The significance level for this study was set at *p* = 0.05. Due to the small sample size, statistical analyses were not performed on the ESD+IPR group. Only demographic statistics will be reported for the ESD+IPR group.

## Results

### Patients Characteristics

**Figure 1:**
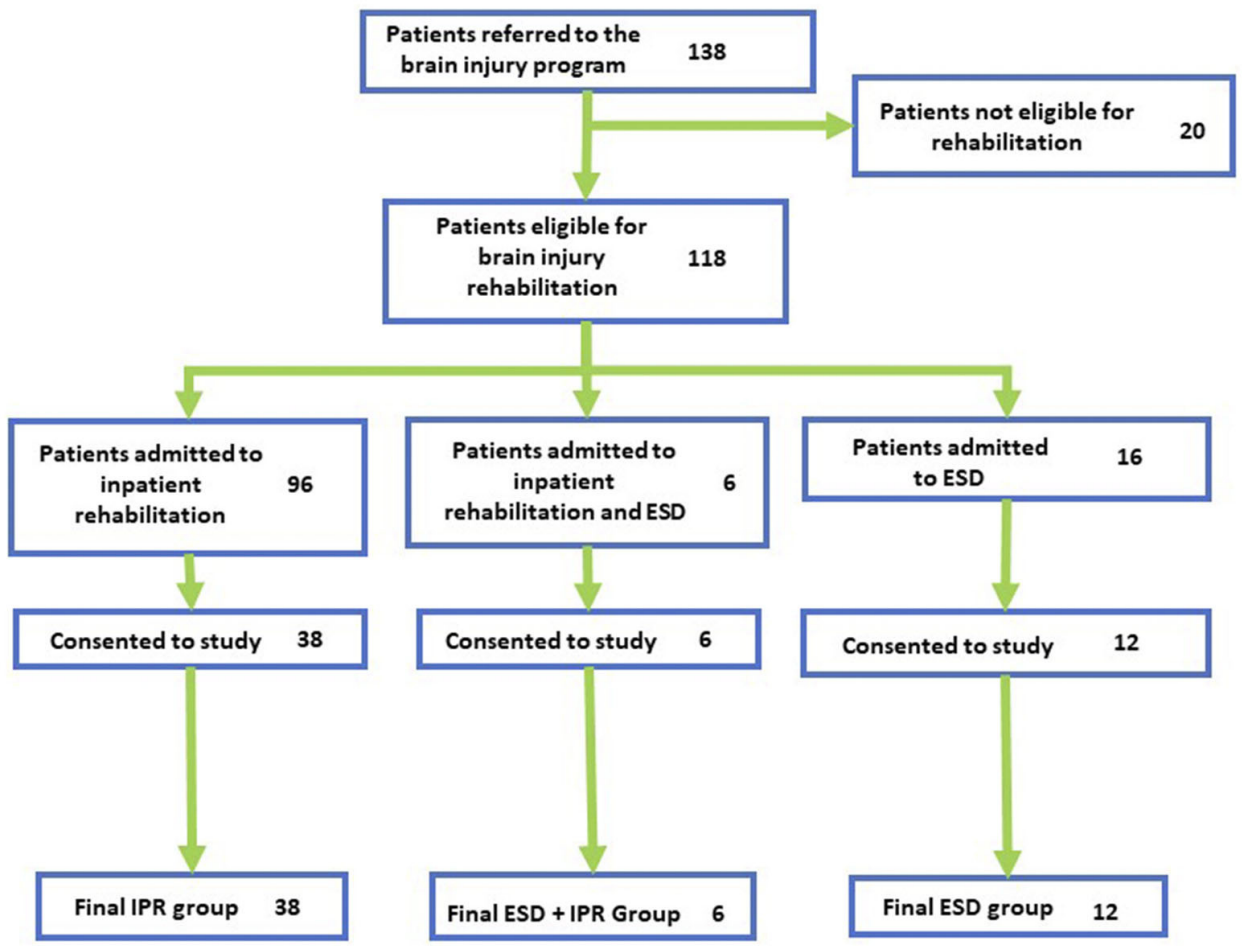
Participant recruitment flow. Flowchart of participants at each stage of the recruitment process. Final groups show participants who completed at least one timepoint.

A flowchart of the patients admitted to ABI neurorehabilitation can be found in Figure 1. One hundred and thirty-eight patients were referred, and 111 were admitted for intensive neurorehabilitation. The IPR unit received 89 patients for neurorehabilitation, of whom 38 agreed to the study. Twenty-two patients were placed into ESD, of whom 18 were recruited (in which six participants participated in both ESD and IPR, these participants were evaluated separately from the other two groups). We were unable to capture all the patients that were admitted to inpatient therapy for a variety of reasons including: patient did not consent, unable to find proxy consent, did not meet initial criteria, and loss to follow-up. Patients that were admitted to IPR but did not participate in the study were not different from the consented IPR patients in terms of their gender (23.4% female; $\chi_{\left(1\right)}^{2}$ = 1.26, *p* = 0.262), age (mean: 49.83, SD: 16.76; *W* = 1,089.00, *p* = 0.665), or type of injury ($\chi_{\left(4\right)}^{2}$ = 1.63, *p* = 0.804); therefore providing a good representation of the IPR cohort.

Demographic information for the IPR, ESD, and IPR+ESD of the groups can be found in Table 1. Groups were not significantly different in mean age *F*_(2, 45)_ = 0.145, *p* = 0.865, or sex $\chi_{\left(2\right)}^{2}$ = 2.463, *p* = 0.292. DRS scores were significantly lower at baseline in the IPR group compared with the ESD group *F*_(2, 45)_ = 3.533, *p* = 0.0368. SRS scores were significantly higher in the IPR group at baseline compared to the ESD group *F*_(2, 45)_ = 18.12, *p* < 0.001. Injury characteristics can be found in Table 2.

**Table 1:** Patient characteristics.

	**Rehabilitation Group**		
	**ESD**	**IPR**	**ESD + IPR**
Age	51.3 (±15.99)	48.47 (±15.98)	42.17 (±17.11)
Sex	33.3% F	36.8% F	50.0% F
**Past medical history**			
Hypertension	5 (41.7%)	9 (24.3%)	1 (20.0%)
Mental health disorders	4 (10.8%)	14 (37.8%)	1 (20.0%)
Headaches	0 (0.0%)	1 (2.7%)	0 (0.0%)
Neurological conditions*	3 (8.1%)	0 (0.0%)	0 (0.0%)
**Education**			
< Grade 12	0 (0.0%)	6 (16.2%)	0 (0.0%)
Grade 12	2 (16.7%)	9 (24.3%)	2 (40.0%)
Trades/vocational	1 (8.3%)	6 (16.2%)	0 (0.0%)
Bachelor's degree	3 (25%)	12 (32.4%)	3 (60.0%)
Master's degree	2 (16.7%)	0 (0.0%)	0 (0.0%)
PhD/medical doctorate	1 (8.3%)	1 (2.7%)	0 (0.0%)
**Employment**			
Employed	3 (25%)	8 (21.6%)	2 (40.0%)
Unemployed	8 (66.7%)	22 (59.4%)	3 (60.0%)
**Marital status**			
Married	7 (58.3%)	18 (48.6%)	2 (33.3%)
Common law	1 (8.3%)	1 (2.7%)	1 (16.7%)
Divorced	1 (8.3%)	4 (10.8%)	0 (0.0%)
Widowed	1 (8.3%)	2 (5.4%)	0 (0.0%)
Single	1 (8.3%)	12 (32.4%)	2 (33.3%)

* *Neurological conditions include all neurological disorders such as: dementia, migraines, epilepsy, paraplegia, and hemiplegia*.

**Table 2:** Injury and rehabilitation characteristics.

	**Rehabilitation Group**		
	**ESD**	**IPR**	**ESD + IPR**
Time since injury for first assessment	29.9 (±46.31)	23.28 (±27.67)	19.75 (±15.66)
Time in acute care	29.1 (±37.05)	33.5 (±26.87)	26.0 (±12.82)
Admission to ICU	1 (8.3%)	21 (56.8%)	1 (16.7%)
Time in ICU	10 (±0.0)	12.1 (±9.35)	22 (±0.0)
Time spent in IPR	0	50.03(±31.37)	61.17 (±45.65)
Time spent in ESD	51.2 (±22.98)	0	89.67 (±12.19)
**Type of injury**			
Traumatic	3	22	2
Cancer	5	6	1
HIE	2	8	0
Epilepsy surgery	1	1	1
Other	0	2	2
**Medical complications**			
Infection of fever	2	16	0
Emotional issues	1	7	0
Skin ulcers	1	1	0
DVT	0	1	0

*The ABIs that fell under the “other” category included: radiation necrosis, HSV-1 encephalitis, left middle cerebral artery aneurysm rupture, and intracranial hypotension*.

The number of days spent in the different services and medical complications are presented in Table 2. Patients in the IPR group had more admissions to intensive care than those in ESD or ESD + IPR groups. Time spent in acute care was similar across groups *F*_(2, 45)_ = 0.234, *p* = 0.792. There were no differences in time between injury and first assessment, between groups (Table 2). The proportion of patients that had a medical complication during rehabilitation was 25.3% in ESD compared to 74.7% patients in IPR with infections, fevers, and mental health difficulties being the most common. No medical complications were reported in the ESD + IPR group.

### Outcome Measures

Functional outcome measures for each group are provided in Tables 3–5. The professionally-rated MPAI total score did not have a significant effect of time in the ESD group ($\chi_{\left(2\right)}^{2}$ = 1.160, *p* = 0.0560) or in the IPR group ($\chi_{\left(2\right)}^{2}$ = 4.750, *p* = 0.093). The professionally-rated MPAI ability sub-score also did not show a significant effect of time in either ESD or IPR groups ($\chi_{\left(2\right)}^{2}$ = 0.204, *p* = 0.903; $\chi_{\left(2\right)}^{2}$ = 2.436, *p* = 0.296). Age did not significantly influence the model for either group. The professionally-rated MPAI adjustment sub-score was not significant in the ESD ($\chi_{\left(2\right)}^{2}$ = 0.111, *p* = 0.946) or IPR groups ($\chi_{\left(2\right)}^{2}$ = 0.712, *p* = 0.700). The professionally-rated MPAI participation sub-score with *post-hoc* Bonferroni adjustment showed significant improvement between 1 and 3 month timepoints in the ESD group [$\chi_{\left(2\right)}^{2}$ = 42.429, unadjusted *p* < 0.000, (Bonferroni adjusted *p* = 0.007)] or IPR group [$\chi_{\left(2\right)}^{2}$ = 9.773, *p* = 0.008, unadjusted (*p* < 0.001)].

**Table 3:** Outcome measure for patients participating in ESD.

		**Post injury**		
	**Initial assessment**	**1 Month**	**3 Month**	**6 Month**
DRS	1.82 (±2.04)	1.58 (±1.75)	1.0 (±1.35)	1.0 (±1.68)
SRS	2.5 (±2.11)	2.0 (±1.78)	1.67 (±1.37)	1.83 (±1.77)
Zarit burden scores	–	**6.67 (±3.8)**	1.0 (±1.41)	1.43 (±2.13)
MoCA	24.8 (±3.03)	25.5 (±3.2)	26.33 (±3.16)	25.8 (±3.31)
PHQ9	2.11 (±1.37)	5.73 (±3.79)	3.67 (±3.06)	3.33 (±2.46)
GAD7	4.9 (±3.86)	4.17 (±4.08)	4.92 (±4.63)	3.83 (±4.0)
QOILBRI	–	71.01 (±10.99)	73.24 (±7.40)	75.74 (±11.09)
**Professionally-rated MPAI**				
Total	–	9.917 (±2.25)	8.417 (±2.58)	8.917 (±3.32)
Participation	–	**5.45 (±1.11)**	1.98 (±0.75)	4.0 (±1.65)
Adjustment	–	2.91 (0.86)	3.17 (±1.34)	3.24 (±0.98)
Ability	–	3.17 (±3.17)	3.41 (±1.13)	2.75 (±1.11)

*Zarit scores were significant in the ESD group for an effect of time with $\chi_{\text{(2)}}^{\text{2}}$ = 31.252, p < 0.001, p < 0.001 with significantly higher scores at the 1 month timepoint than the 3 month, and 6 month timepoints. The MPAI participation subscore was also significantly higher in the 1 month timepoint than the 3 month timepoint ($\chi_{\text{(2)}}^{\text{2}}$ = 42.429, p < 0.000). The bold values indicate significance of p > 0.05*.

**Table 4:** Outcome measures for patients participating in IPR.

		**Post injury**		
	**Initial assessment**	**1 Month**	**3 Month**	**6 Month**
DRS	5.55 (±4.66)	4.18 (±4.24)	2.62 (±2.5)	2.5 (±3.53)
SRS	6.95 (±2.05)	4.46 (±2.77)	2.58 (±1.55)	2.0 (±1.26)
Zarit burden scores	–	16.1 (±12.2)	12.3(±10.72)	10.81 (±10.19)
MoCA	18.85 (±5.28)	21.36 (±6.45)	**23.11 (±4.84)**	**25.58 (±3.2)**
PHQ9	2.38 (±1.89)	**5.68 (±5.22)**	**5.58 (±5.07)**	**6.8 (±5.05)**
GAD7	7.44 (±5.24)	5.48 (±5.12)	5.19 (±4.76)	4.63 (±4.56)
QOLIBRI	–	66.60 (±16.37)	61.46 (±16.55)	66.64 (±18.41)
**Professionally-rated MPAI**				
Total	–	30.83 (±4.15)	20.19 (±5.52)	21.31 (±3.47)
Participation	–	**14.48 (±1.55)**	8.82 (±1.35)	9.16 (±1.46)
Adjustment	–	9.29 (±1.74)	8.08 (±1.53)	7.33 (±1.48)
Ability	–	10.5 (±2.08)	6.96 (±1.46)	7.82 (±1.54)

*The MPAI participation sub-score improved over time and had a significant effect of time in the IPR group ($\chi_{\text{(2)}}^{\text{2}}$ = 9.773, p = 0.008), showing significantly increased scores in the 1 month timepoint compared with the 3 month timepoint. The MoCA significantly improved over time ($\chi_{\text{(2)}}^{\text{2}}$ = 22.902, p < 0.001), between both 1 and 3, and 1 and 6 month timepoints. The PHQ9 scores improved over time as well ($\chi_{\text{(2)}}^{\text{2}}$ = 18.921, p < 0.001), showing lower scores following Bonferroni correction at baseline compared to 1 month (p = 0.004), 3 months (p < 0.001), and 6 month (p = 0.005). The bold values indicate significance of p > 0.05*.

**Table 5:** Outcome measures for patients participating in ESD + IPR.

		**Post injury**		
	**Initial assessment**	**1 Month**	**3 Month**	**6 Month**
DRS	4.0 (±0.89)	2.75 (±1.3)	1.67 (±1.25)	1.67 (±0.94)
SRS	4.8 (±1.94)	3.5 (±1.12)	2.0 (±0.0)	2.67 (±1.7)
Zarit Burden Scores	–	17.5 (±3.5)	16.5 (±2.5)	12.67 (±5.79)
MOCA	20.75 (±5.8)	19.6 (±5.24)	18.0 (±2.0)	16.5 (±3.5)
PHQ9	2.2 (±3.12)	2.0 (±1.63)	6.67 (±7.32)	2.0 (±0.5)
GAD7	2.0 (±1.63)	4.5 (±3.57)	2.5 (±0.5)	3.0 (±1.41)
QOLIBRI	–	83.11 (±1.35)	61.83 (±15.88)	51.01 (±13.85)
**Professionally-rated MPAI**				
Total	–	19.63 (±3.3)	33.13 (±13.2)	26.63 (±6.7)
Participation	–	7.59 (±1.52)	14.47 (±4.02)	10.85 (±2.52)
Adjustment	–	5.71 (±1.17)	13.21 (±6.62)	7.71 (±2.08)
Ability	–	9.06 (±1.88)	12.44 (±5.76)	10.31 (±3.31)

ZBI scores with Bonferroni correction were significantly higher at 1 month compared to 3, and 1 month compared to 6 months in the ESD group ($\chi_{\left(2\right)}^{2}$ = 31.252, *p* < 0.001), (*p* < 0.001). ZBI scores did not significantly change over time for the IPR ($\chi_{\left(2\right)}^{2}$ = 1.482, *p* = 0.477) group. Age was not influential in either group (Tables 3–5). The MoCA had a significant effect of time in the IPR group ($\chi_{\left(2\right)}^{2}$ = 22.902, *p* < 0.001) with Bonferroni correction showing significantly lower scores in the baseline measurement compared to the 3 month (*p* = 0.003) and 6 month (*p* < 0.001). The MoCA did not significantly change over time in the ESD group ($\chi_{\left(2\right)}^{2}$ = 4.206, *p* = 0.240). The QOLIBRI was not significant over time in either IPR or ESD groups ($\chi_{\left(2\right)}^{2}$ = 4.457, *p* = 0.108; $\chi_{\left(2\right)}^{2}$ = 2.979, *p* = 0.225). The GAD7 was not significant over time in either IPR or ESD groups ($\chi_{\left(2\right)}^{2}$ = 0.915, *p* = 0.633; $\chi_{\left(2\right)}^{2}$ = 1.099, *p* = 0.577). The PHQ9 scores significantly increased in the IPR group ($\chi_{\left(2\right)}^{2}$ = 18.921, *p* < 0.001), showing lower scores following Bonferroni correction at baseline compared to 1 month (*p* = 0.004), 3 months (*p* < 0.001), and 6 month (*p* = 0.005) timepoints. Age did not influence the model. There was no significant difference in PHQ9 in the ESD group ($\chi_{\left(2\right)}^{2}$ = 6.783, *p* = 0.079).

## Discussion

This study aimed to characterize and evaluate the efficacy of an ESD program for patients with non-stroke ABI. We found all three groups that were followed: IPR, ESD, and ESD + IPR had improvements in function over the 6 month follow-up. However, we were not able to evaluate patients participating in the ESD + IPR group statistically, as the sample size was underpowered. Both ESD and IPR groups had a significant improvement in the participation sub-score on the professionally-rated MPAI-4. No significant changes were observed in either group in the MPAI total score, or the ability and adjustment sub-scores. Notably, caregiver burden of patients in the ESD and ESD + IPR groups improved throughout the study, with the ESD group showing significant improvement. We found the MoCA and PHQ9 significantly increased over time in the IPR group but not the ESD group, signifying an improvement in cognition over time, with worsening depressive symptoms. We found no significant changes in quality of life or anxiety over time in either group. Additionally, we found there were fewer medical complications in patients with ABI participating in ESD compared to IPR group, with no complications in the ESD + IPR groups. While not explicitly evaluated in this study, previous literature in the stroke population indicates that ESD programs are far more cost-effective than inpatient rehabilitation (5). The economic advantage provides an important rationale for comparison of rehabilitation streams.

### Patient Selection

Based on DRS scores, our physiatry team referred more severely impaired patients with ABI to IPR than to ESD or ESD + IPR groups, creating distinctly different groups. This choice was functionally appropriate, as patients who demonstrated higher disability scores were more suited for inpatient care throughout recovery. As the groups were clearly different patient populations due to severity of disability, we could not directly compare them for this study. The difference in patient disability between the IPR and ESD group shows that an ESD neurorehabilitation program may not be appropriate for all patients with ABI. However, for patients with mild-to-moderate impairment requiring intensive rehabilitation an ESD program may be more efficacious model.

### Functional Outcomes and Caregiver Burden

We selected the MPAI-4 and its sub-scores to assess function as it has been shown to be a reliable measure for overall function in the patients with brain injury (17). Both ESD and IPR groups showed improvements in total score as well as ability, adjustment, and participation sub-scores of the MPAI with statistically significant improvements noted in the participation sub-score of both groups. The components involved in tallying the participation score included: relationships with family and significant others, initiating social activities, participation in leisure activities, and self-care (hygiene, dressing, etc.). Improvement in both groups indicates that regardless of location, both rehabilitation streams are improving in similar domains of recovery, with parallel results. The small sample size of the ESD + IPR group prevented appropriate statistical comparison, however, this group demonstrated robust improvements in MPAI total score, as well as each sub-score. This group may represent a population that maximized an effective transition from inpatient to ESD rehabilitation. Our results are similar to ESD studies completed in stroke populations. For example, a randomized control trial of ESD vs. standard of care for patients with moderately disabled stroke was completed by Thorsén et al. (10). They found similar improvements in motor capacity, manual dexterity, ADLs, and health related quality of life at 3 months post-stroke for both inpatient and ESD rehabilitation streams, concluding that ESD for moderately-disabling stroke is as effective as standard inpatient rehabilitation.

Perceived caregiver burden, measured by the Zarit burden Interview (ZBI), significantly improved throughout recovery in the ESD group but not in the IPR group. Lower scores indicate that caregivers of the ESD group reported feeling less stressed and better adjusted than caregivers of patients in the IPR group. Perceived burden may have been higher in the IPR group because of increased trips to the hospital, fees associated with hospital visits, as well as the notion that their loved one is not well-enough to leave hospital. Previous research in the stroke population found reduced caregiver burden when rehabilitation was completed in the home setting (24). The results of our study were similar, suggesting that caregivers experience reduced burden when their loved ones are in an ESD rehabilitation stream, compared to inpatient rehabilitation.

As we aimed to describe the groups separately, many similarities were evident in their recovery trajectories. All groups demonstrated improvement across the MPAI and its sub-scores, regardless of location during rehabilitation. Improvement over time was also evident in GAD7, MoCA, and QOLIBRI scores in all groups. In short, patients showed successful recovery in all rehabilitation streams. These findings are encouraging as they support a model that incorporates varying types of rehabilitation, focused on the individual needs of the specific patient.

### Affective and Quality of Life Outcomes

Despite many similarities to stroke recovery, patients recovering from non-stroke ABI can present with unique affective and behavioral symptoms that may impact the feasibility of an ESD rehabilitation program. Posttraumatic aggression has been well-documented both scientifically and anecdotally but is often not considered in the context of neurorehabilitation (1, 2, 25). Therefore, a patient who may otherwise be an ideal candidate for ESD may benefit from inpatient care in order to manage aggression or unpredictable behavior. By the same token, a patient struggling to thrive in an inpatient setting due to problems with motivation or stamina may be experiencing affective symptoms (i.e., depression or anxiety) that could be managed more effectively in the home environment. Our study demonstrated a reduction in anxiety in the ESD + IPR group only, with no significant changes over time in the IPR or ESD groups. As well, PHQ9 scores in the IPR group significantly increased throughout recovery, whereas there was little change in depressive scores in the ESD group. Increased depressive symptoms seen in the IPR group may reflect increased frustration and awareness of personal limitations as they remain in hospital. The IPR group also had decreased quality of life, whereas the ESD group did not, highlighting an important advantage of ESD programs, as they may allow the patient to feel more independent and fulfilled if able to be at home. As these results are interesting, but preliminary, a well-powered study is required to properly elucidate the relationship between rehabilitation stream and changes to affective symptoms and quality of life.

### Medical Complications

The number of medical complications experienced during rehabilitation by patients participating in ESD was less than in patients participating in IPR, and absent in those ESD + IPR patients. While these differences are clinically meaningful, our study was underpowered to determine if those differences were statistically meaningful. In the current study, the most common complications were infections, fevers, and mental health difficulties. Medical complications such as infections and mental health difficulties can negatively influence rehabilitation participation and potentially prolong recovery. The reason for additional medical complications in the IPR group most likely is multifactorial. Patients in the IPR group had a greater DRS suggesting they were more severely impaired and potentially more likely to have complications. As well, there may be an increase risk of contacting infection from other patients on an inpatient ward compared to home. Finally, extended hospital stays have the potential to negatively impact mental health. Providing rehabilitation in the home may decrease infections and mental health difficulties and be a more efficient avenue to see improvements from intensive rehabilitation, and thereby potentially improve patient care.

### Strengths and Limitations of the Study

There are many strengths of this study. All patients were enrolled in the study when they met criteria for intensive neurorehabilitation, despite the severity of the initial injury. We were then able to accurately measure functional change due to the type of rehabilitation received, as all patients were able to engage in therapies at an appropriate level. Another strength of the study is that we were able to collect data at multiple time points. Patients were followed for 6 months following readiness for rehabilitation and data was collected at four time points during that period. Finally, this study looked at multiple areas that are important to rehabilitation such as functional outcomes, patient mental health, and quality of life, caregiver burden, and medical complications.

However, this patient population and study design has inherent limitations. Patients were not randomized nor was the research team blinded to rehabilitation stream, causing inherent risk of observer bias. However, all patients chosen to participate in the study met the criteria for participation in a neurorehabilitation program and those choosing the rehabilitation stream were not active research team members in the study. As well, during the study, some patients were lost to follow-up at various time points and therefore we do not have a complete data set at all follow-up time points. Furthermore, the patient population studied was very heterogeneous. This reflects the types of ABI our facility admits for intensive neurorehabilitation, and most likely mirrors other facilities across Canada.

## Conclusions

Heterogeneity and the small sample size limit our ability to draw specific conclusions for a certain type of ABI, but does reflect current clinical practice. In conclusion, this study describes the possibility that ESD-type programs may be implemented for the appropriately selected patient group with ABI. Functional improvement, less caregiver burden, and fewer medical complications highlight the strengths of having an ESD neurorehabilitation stream. Future studies, using a more robust study design such as a randomized control trial and a larger sample size are warranted to further evaluate ESD as an intensive neurorehabilitation option for patients with ABI.

## Data Availability Statement

The raw data supporting the conclusions of this article will be made available by the authors, without undue reservation.

## Ethics Statement

The studies involving human participants were reviewed and approved by University of Calgary Research Ethics Board. The patients/participants provided their written informed consent to participate in this study.

## Author Contributions

RK contributed to data analysis, manuscript preparation, and manuscript review. TSe contributed to data collection, data analysis, and manuscript preparation. MW, TSa, and TF contributed to data analysis and manuscript review. LP and JK contributed to study design and manuscript review. RL contributed to study design, data collection, and manuscript review. CD contributed to study design, data collection, manuscript preparation, and manuscript review. All authors contributed to the article and approved the submitted version.

## Conflict of Interest

The authors declare that the research was conducted in the absence of any commercial or financial relationships that could be construed as a potential conflict of interest.
